# Dietary Supplementation with Methylsulfonylmethane and Myo-Inosito Supports Hair Quality and Fecal Microbiome in Poodles

**DOI:** 10.3390/ani14243643

**Published:** 2024-12-17

**Authors:** Jie Zhang, Dan Guo, Limeng Zhang, Deping Li, Baichuan Deng

**Affiliations:** 1 Guangdong Provincial Key Laboratory of Animal Nutrition Control, College of Animal Science, South China Agricultural University, Guangzhou 510642, China; zjie1103@stu.scau.edu.cn (J.Z.); guod@petholding.com (D.G.); 2 Guangzhou Qingke Biotechnology Co., Ltd., Guangzhou 510000, China; 19865040001@163.com; 3 Hangzhou Netease Yanxuan Trading Co., Ltd., Hangzhou 310051, China; lideping@corp.netease.com

**Keywords:** methylsulfonylmethane, myo-inositol, hair quality, fecal microbiota, poodle

## Abstract

This study aimed to investigate the effects of dietary supplementation with methylsulfonylmethane (MSM) and myo-inositol (MI) on hair quality, fecal microbiota, and metabolome in poodles. Thirty-two adult poodles categorized based on initial body weight and sex were randomly assigned to four groups. These groups (designated the CON, MSM, MI, and MSM + MI groups) received a basal diet, the same diet supplemented with 0.2% MSM + 0% MI, the same diet supplemented with 0% MSM + 0.2% MI, or the same diet supplemented with 0.2% MSM + 0.2% MI, respectively. The study lasted for 65 days. During the entire study period, body weight, average daily weight gain, feed intake, energy intake, and fecal output were normal in all the animals and did not differ significantly among the treatment groups. Hair scale thickness was lower in the MI and MSM + MI groups than in the CON group on Day 65 (*p* < 0.05). An amino acid analysis of the hair revealed higher sulfur content in the MI and MSM + MI groups on Day 65 than on Day 0 (*p* < 0.05). Moreover, the poodles in the MSM, MI, and MSM + MI groups presented significantly lower levels of *Proteobacteria_unclassified* and *Candidatus Phytoplasma* than did those in the CON group. The relative abundance of *Gammaproteobacteria_unclassified* was greater in the MSM and MI groups than in the CON group (*p* < 0.05). The MSM group presented a greater abundance of *Glucerabacter* than the CON group (*p* < 0.05). Compared with those in the CON and MSM + MI groups, the abundances of *Paramuribaculum* and *Hafnia* in the MSM group were greater (*p* < 0.05). The abundances of *Enterobacter* and *Kineothrix* were greater (*p* < 0.05) in the MI group than in the CON and MSM + MI groups. The poodles in the MI group presented significantly greater abundances of *Bacteroidales_unclassified*, *Halanaerobium*, *Mycobacterium*, and *Erysipelotrichaceae_unclassified* than did poodles in the CON, MSM, and MSM + MI groups. Fecal metabolomics analysis revealed that MSM, MI, and MSM + MI treatment markedly affected carbohydrate metabolism. MSM + MI treatment also influenced lipid metabolism. These findings suggest that dietary supplementation with MSM and MI can improve the hair quality of poodles.

## Simple Summary

Methylsulfonylmethane (MSM) has been used as a supplement for skin nourishment, hair growth promotion, and hair quality improvement. Myo-inositol (MI) plays a role in various physiological processes in animals. The aim of this study is to assess the effects of MSM and MI as feed additives on the hair quality of poodles. Compared to the control, supplementation with MSM and MI decreased scale thickness, increased the contents of hair sulfur, and regulated gut microbiota and fecal metabolites. In conclusion, MSM and MI enhanced hair smoothness, suggesting potential benefits to fecal microbiota and the metabolic profiles of poodles. In conclusion, MSM and MI supplementation enhanced hair smoothness and appeared to positively influence fecal microbiota composition and metabolic profiles in poodles.

## 1. Introduction

Functional ingredients have been widely used in pet food to improve the health conditions of companion animals by relieving stress, fighting obesity and improving gut homeostasis [1,2]. The largest connective tissues in dogs are skin and hair; these account for approximately 14–26% of a dog’s total weight [3]. The hair coat plays an important role in protecting the skin from ultraviolet light and physical damage and in regulating body temperature [4]. Pet owners often view the hair quality of animals as a fundamental indicator of the nutritional benefits of their pet food [5]. Dog coat health is affected by disease, nutrition, drugs, and the environment. A study of beagles revealed that diets containing increased amounts of polyunsaturated fatty acids can improve hair coat glossiness and softness [6]. Another study demonstrated the beneficial effects of supplementation of the diet with both zinc and linoleic acid on the hair coat condition of dogs [7]. These advances in understanding the nutrition required to produce optimal hair coats in dogs support a continued desire to investigate factors that influence hair coat quality in dogs.

Methylsulfonylmethane (MSM), also known as dimethylsulfone, is a naturally occurring organic sulfur-containing compound that is widely present in animals and humans [8]. The benefits of MSM include its anti-inflammatory properties [9], its antioxidant capacity [10], and its antitumor effects [11]. As a result, MSM is commonly used as a health supplement or therapeutic agent in humans. The U.S. Food and Drug Administration recognizes MSM as a natural source of sulfur that can be used as a dietary supplement to improve joint health [12]. The evidence also suggests that MSM is beneficial for skin nourishment, growth promotion, and hair quality improvement. For example, MSM was shown to nourish human skin [13], promote growth in broilers [14] and enhance hair growth in mice [15]. Our previous study demonstrated the beneficial effects of MSM on promoting hair quality in kittens [16].

Myo-inositol (MI), also known as cyclohexanehexol, plays a role in various physiological processes in animals [17]. MI is widely distributed in animals, where it is a component of membrane phospholipids and mediates osmoregulation [18]. Its phosphorylated derivatives act as second messengers in signal transduction pathways [19], mediate the phosphorylation of proteins [20], participate in chromatin remodeling and gene expression [21,22], and facilitate export of mRNA from the nucleus [23]. In addition, Woolley reported that inositol has a therapeutic effect on hair deficiency in mice [24]. MI has been demonstrated to exert a plethora of valuable health effects [25].

Although studies of the effects of MSM and MI on the hair of other animals have been conducted, research on their impact and their potential mechanisms of action in canines is still lacking. Methylsulfonylmethane is used to nourish skin, hair, and fingernails due to its sulfur concentration [26]. In a previous study, myo-inositol derivative was expressed in a subtype-specific manner in hair follicles and regulated the hair cycle [27]. We assumed that mixing two substances with different principles of action will give better results. The aim of this study was to assess the effects of MSM and MI as feed additives on hair quality, the fecal microbiota, and the metabolome in an animal model (poodles).

## 2. Materials and Methods

### 2.1. Ethical Considerations

All experimental procedures were authorized by the Animal Care and Use Committee prior to animal experimentation (Approval number 2021E028) and were performed according to the guidelines provided by the Laboratory Animal Center at South China Agricultural University. The husbandry and housing of the animals conformed to the recommendations set forth in the Animal Welfare Act and in the Guide for the Care and Use of Laboratory Animals (8th ed.) [28].

The poodles used in this study were housed in an animal room 60 square meters in size. The room was maintained at a constant temperature and humidity (23 °C, 70%) with a 12 h light/dark cycle. The room was cleaned and disinfected once a day. An environmentally enriched open area 50 square meters in size was provided for group socialization, walks, and exercise for each poodle ad libitum. All the poodles experienced much contact with humans during the day.

The poodles were given fresh water ad libitum throughout the trial. Two daily observational health and well-being checks and monthly veterinary assessments were performed during the study. Vaccination and deworming were administered prior to the study, and the poodles did not receive any drugs that cause changes in the intestinal microflora (e.g., antibiotics) during the month prior to the study.

### 2.2. Experimental Design, Animals, and Management

#### 2.2.1. Animals and Diets

In this study, miniature poodles (15–19 inches in length) were used as model animals. A total of 32 adult poodles (average age 3.5 ± 1.14 years) were randomly assigned according to sex and initial body weight (BW) to 1 of the 4 dietary treatment groups, each of which included 8 animals (4 females and 4 males). The diets received by the animals included the following: (1) a basal diet (CON group); (2) the basal diet supplemented with 0.2% MSM (MSM group); (3) the basal diet supplemented with 0.2% MI (MI group); and (4) the basal diet supplemented with 0.2% MSM and 0.2% MI (MSM + MI group). The diets were manufactured at Guangzhou Qingke Biotechnology Co., Ltd. (Guangzhou, China) and were formulated to meet the nutritional requirements of adult poodles recommended by the Association of American Feed Control Officials [29]. The four experimental diets had the same ingredient composition except for the absence or inclusion of MSM and MI. The ingredients and nutrient levels of the experimental diet are presented in Table 1.

#### 2.2.2. Experimental Design

After at least one month of acclimation to the environment, the poodles were randomly assigned to 4 experimental groups. The study lasted for 65 days, including a 5-day wash-out period and a 60-day experimental period. A restricted diet of 80 g per poodle was offered at each of the two daily meals, which were given at 8:30 am and 5:30 pm. During feeding, all dogs were housed individually in cages (0.7 × 0.7 × 0.8 m) to allow for the monitoring of their food consumption. Serum biochemistry analysis and weighing were performed before the morning feeding on Day 0 (i.e., 1 day before the trial). Whole feces were collected on Days 60 to 64. BW and hair score (HS) were recorded on Day 65 before the morning feeding, and fresh feces and blood samples were collected on Day 65 for further analysis.

### 2.3. Chemical Analysis of Diets and Feces

To measure the apparent total tract digestibility of nutrients, samples of the four experimental diets and feces from the animals were collected and stored in a freezer at −20 °C. The samples were then oven-dried at 65 °C for 48 h to constant weight, finely ground, and passed through a 1 mm mesh screen. The dry matter and organic matter contents of the diet and of the fecal samples were determined according to the methods of the Association of Official Analytical Chemists [31]. Crude protein, total dietary fiber, and acid-hydrolyzed fat were analyzed via the Kjeldahl method using a semiautomatic Kjeldahl apparatus (VAPODEST 200, C. Gerhardt GmbH & Co. KG, Königswinter, Germany), an automatic fiber analyzer (FIBRETHERM FT12, C. Gerhardt GmbH & Co. KG, Germany), and a fatty analyzer (FT640, Grand Analytical Instrument Co., Ltd., Guangzhou, China). The GE was analyzed using an oxygen bomb calorimeter (IKA C 200, IKA (Guangzhou) Instrument Equipment Co., Ltd., Guangzhou, China). Finally, the digestibility of nutrients was calculated via the following formula: Nutrient digestibility (%) = [(Nutrient intake − Nutrient in feces)/Nutrient intake] × 100% (g/d, DM basis).

### 2.4. Serum Biochemistry and Inflammatory Cytokine Analyses

On Day 0 and Day 65, after overnight fasting, 5 mL blood samples were harvested from the forelimb vein of each poodle. The blood was transferred to a precooled serum separator tube and allowed to stand for 30 min before centrifugation at 3500× *g* at room temperature for 15 min. The supernatants of this centrifugation were collected as serum samples. The obtained serum was used to measure blood biochemical parameters using an automatic biochemical analyzer (SMT120VP, Chengdu Seamaty Technology Co., Ltd., Chengdu, China). The serum contents of interferon-γ, interleukin-1β, immunoglobin G, interleukin-8, and tumor necrosis factor were measured using commercial canine ELISA kits (MEIMIAN, Jiangsu Meimian Industrial Co., Ltd., Yancheng, China).

### 2.5. Hair Growth and Quality Analysis

**Figure 1:**
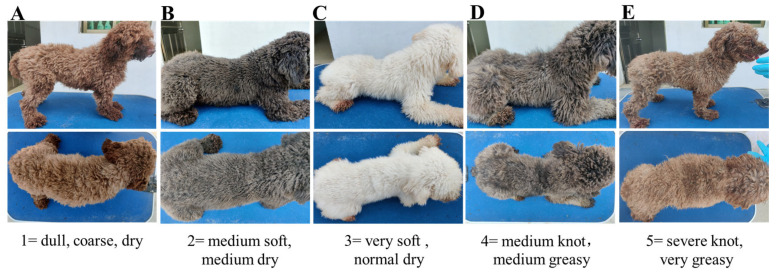
Hair rating criteria for poodles fed MSM- and MI-supplemented diets. All samples were scored in 5 increments using the following scale: (**A**) 1 = dull, coarse, dry; (**B**) 2 = medium soft, medium dry; (**C**) 3 = very soft, normal dry; (**D**) 4 = medium knots, medium greasy; and (**E**) 5 = severe knots, very greasy.

The hair on the backs of all the poodles was shaved on Day 0, and the length of the new hair at the same position was measured on Day 65. Hair scoring was conducted on Day 65 under consistent lighting conditions on the same observation table. The seven specially trained observers were blinded to the treatments received by the individual poodles. The method described by Rees [32] and Guo Dan [16] was adopted for the evaluation of hair scores. Briefly, all samples were scored in 5 increments using the following scale: 1= dull, coarse, dry; 2 = medium soft, medium dry; 3 = very soft, normal dry; 4 = medium knots, medium greasy; 5 = severe knots, very greasy. A score of 1 or 2 was considered to indicate “dry”; a score of 3 was considered “ideal”, and a score of 4 or 5 was considered to indicate “greasy” (Figure 1). On Days 0 and 65, hair samples were collected for measurement of scale structure and S content. The structural characteristics of the scale layer, including the thickness and height of the scale and the hair diameter, were measured by scanning electron microscopy (FBI-SEM, LYRA3XMH, TESCAN, Brno, Czech Republic). In addition, the S content of the samples was measured via semiquantitative detection analysis using an X-ray energy spectrometer (X-MaxN, Oxford Instruments, Oxford, UK).

### 2.6. Fecal Microbiota Analysis

On Day 65, fresh fecal samples were collected within 15 min of defecation and stored in a freezer at −80 °C for microbiota analyses. In brief, the total microbial DNA present in the feces was extracted using the E.Z.N.A. @ Stool DNA Kit (D4015, Omega, Inc., Norwalk, CT, USA). A targeted PCR-based sequencing approach was used in which the V3–V4 regions of the 16S rDNA gene were targeted to generate amplicons using the primers 341F (5′-CCTAYGGGRBGCASCAG-3′) and 805R (5′-GACTACHVGGGTATCTAATCC-3′) with barcodes. PCR amplification was performed in a total volume of 25 μL of reaction mixture containing 25 ng of template DNA, 12.5 μL of PCR Premix, 2.5 μL of each primer, and an amount of PCR-grade water needed to adjust the volume. The PCR cycling parameters were 98 °C for 30 s followed by 32 cycles of 98 °C for 10 s, 54 °C for 30 s, and 72 °C for 45 s and a final extension of 72 °C for 10 min. The PCR products were analyzed by electrophoresis on 2% agarose gels, purified using AMPure XT beads (Beckman Coulter Genomics, Danvers, MA, USA), quantified using Qubit (Invitrogen. Waltham, MA, USA), and sequenced on a NovaSeq PE250 platform at LC-Bio Technology Co., Ltd., Hang Zhou, Zhejiang Province, China. The reads were merged via FLASH. Quality filtering of the raw reads was performed to obtain high-quality clean tags according to fqtrim (v0.94). Chimeric sequences were filtered using Vsearch software (v2.3.4). After dereplication using DADA2, the feature table and feature sequence were obtained. Then, according to the SILVA (release 138) classifier, feature abundance was normalized to the relative abundance of each sample. Alpha diversity, including Chao1, observed species, Shannon, and Simpson indices, was calculated using QIIME2. The Chao1 estimator and observed species are the metrics used to estimate the total number of species. The Shannon index and Simpson index are metrics of relative community evenness, reflecting the richness and evenness of the species in a sample [33]. Principal component analysis (PCA) was performed with R software (v3.4.4). nsegata-LEfSe software (094f447691f0) was used to perform LEfSe.

### 2.7. Fecal Metabolomic Analysis

Fecal samples were collected on Day 65 and stored at −80 °C for metabolomic analysis via an untargeted approach using chromatography mass spectrometry. The UPLC–Orbitrap–MS/MS system from Thermo Fisher Scientific (Q Exactive Focus, Waltham, MA, USA) was used as an untargeted metabolomic approach to determine the fecal metabolic profiles [34]. The Compound Discoverer 2.1 (Thermo Fisher Scientific, Waltham, MA, USA) data analysis tool was employed for automated raw data preprocessing and metabolite identification by searching the mzCloud and mzVault libraries. Principal component analysis of the metabolites was conducted. In addition, the variable importance (VIP) in the projection was computed via the OPLS-DA model. The metabolites with VIP > 1 and *p* < 0.05 were deemed differentially abundant metabolites. The Kyoto Encyclopedia of Genes and Genomes (KEGG) database was used to functionally annotate these differentially abundant metabolites, and the metabolites were further mapped to the KEGG pathway database using MetaboAnalyst 5.0.

### 2.8. Statistical Analysis

Statistical analysis was performed using SPSS 26.0 software. One-way analysis of variance followed by the least significant difference multiple range test was used to determine the statistical significance of multiple comparisons in the experiment. For fecal microbial bacterial diversity, the Wilcoxon signed rank test was used as a significance test. Nonparametric Kruskal–Wallis tests were used to compare microbial relative abundance compositions. Graphs were drawn using GraphPad Prism 8.0 software. The mean values were based on 8 replicates per group, and the variability in the data was expressed as the standard error of the mean (SEM). Statistical significance was defined as *p* < 0.05, and a trend was considered for 0.05 ≤ *p* < 0.10.

## 3. Results

### 3.1. Body Weight and Nutrient Digestibility

As shown in Table 2, neither initial (Day 0) or final (Day 65) BW differed among the four treatment groups (*p* > 0.05), and the average daily weight gain of the experimental groups was not significantly different from that of the CON group. Moreover, there were no differences (*p* > 0.05) in feed intake, energy intake, or fecal output among the treatment groups.

As shown in Table 3, the digestibility of acid-hydrolyzed fat was significantly greater in the MSM and MI groups than in the CON group. The addition of MSM or MI had no effect on the nutrient digestibility of dry matter, organic matter, crude protein, crude fiber, or gross energy.

### 3.2. Serum Biochemistry and Inflammatory Cytokine Levels

The serum biochemistry of poodles fed diets containing MSM and MI were within reference ranges for healthy poodles both on Day 0 and on Day 65 (Table 4). The blood glucose levels were higher (*p* < 0.05) in the MSM + MI group than in the CON and MSM groups. MSM supplementation increased albumin and TP levels on Day 65 compared with the levels in the other three groups (*p* < 0.05). The effects of dietary MSM and MI supplementation on serum inflammatory cytokines are presented in Table 5. The poodles in the four groups presented no significant differences in the levels of inflammatory cytokines.

### 3.3. Hair Growth and Quality

In our study, there was no significant difference in hair length among the groups, as shown in Table 6.

The hair scores did not differ significantly among the groups; all the groups received hair scores that indicated very soft and normal dry levels (Table 7).

**Figure 2:**
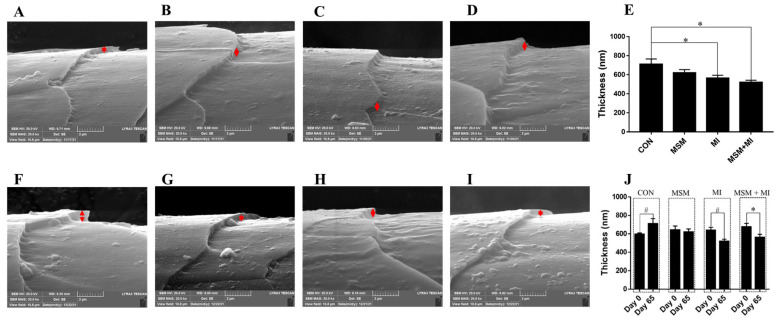
Scale thickness of the hair of poodles fed MSM- and MI-supplemented diets. The scale thickness of the hair of the poodles in the (**A**) CON, (**B**) MSM, (**C**) MI, and (**D**) MSM + MI groups on Day 0. (**E**) The scale thickness of the hair of the poodles in the (**F**) CON, (**G**) MSM, (**H**) MI, and (**I**) MSM + MI groups on Day 65. (**J**) The scale thickness of the hair of the poodles in the four groups on Day 0 and Day 65. CON, basal diet; MSM, basal diet supplemented with 0.2% MSM; MI, basal diet supplemented with 0.2% MI; and MSM + MI, basal diet supplemented with 0.2% MSM and 0.2% MI. The red arrows indicate the scale thickness of the hair. The symbol (*) indicates statistically significant differences between two groups (* *p* < 0.05), and the symbol (#) represents a difference (# 0.05 ≤ *p* < 0.10). The mean values are based on 8 replicates per treatment group and one dog per replicate.

**Figure 3:**
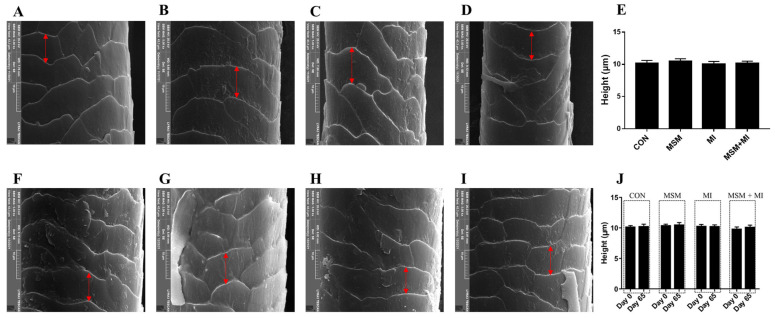
Scale height of the hair of poodles fed MSM- and MI-supplemented diets. Scale height of the hair of the animals in the (**A**) CON, (**B**) MSM, (**C**) MI, and (**D**) MSM + MI groups on Day 0. (**E**) Scale height of the hair of poodles fed diets containing MSM on Day 65. Scale height of the hair of the animals in the (**F**) CON, (**G**) MSM, (**H**) MI, and (**I**) MSM + MI groups on Day 65. (**J**) Scale height of the hair of the poodles in the four groups on Day 0 and Day 65. CON, basal diet; MSM, basal diet supplemented with 0.2% MSM; MI, basal diet supplemented with 0.2% MI; and MSM + MI, basal diet supplemented with 0.2% MSM and 0.2% MI. The red arrows indicate the scale height of the hair. The mean values are based on 8 replicates per treatment group and one dog per replicate.

**Figure 4:**
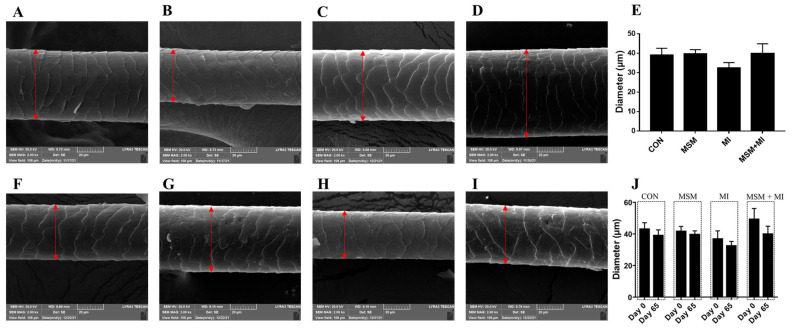
Scale diameter of the hair of poodles fed MSM- and MI-supplemented diets. Scale diameter of the hair of the poodles in the (**A**) CON, (**B**) MSM, (**C**) MI, and (**D**) MSM + MI groups on Day 0. (**E**) Scale diameter of the hair of poodles fed diets containing MSM on Day 0. Scale height of the hair of the poodles in the (**F**) CON, (**G**) MSM, (**H**) MI, and (**I**) MSM + MI groups on Day 65. (**J**) Scale diameter of the hair of the poodles in the four groups on Day 0 and Day 65. CON, basal diet; MSM, basal diet supplemented with 0.2% MSM; MI, basal diet supplemented with 0.2% MI; and MSM + MI, basal diet supplemented with 0.2% MSM and 0.2% MI. The red arrows indicate the scale diameter of the hair. The mean values are based on 8 replicates per treatment group and one dog per replicate.

A significant decrease in hair scale thickness was observed in the MI and MSM + MI groups compared with the CON group on Day 65 (Figure 2). There were no significant differences in hair scale height among the groups (Figure 3). Moreover, no impact of the dietary supplementation on hair diameter was observed in the current experiment, as shown in Figure 4.

Analysis of the composition of the hair revealed that the S content of the hair of the animals in the MI and MSM + MI groups was greater on Day 65 than it had been on Day 0 (*p* < 0.05, Table 8).

### 3.4. Fecal Microbiota

**Figure 5:**
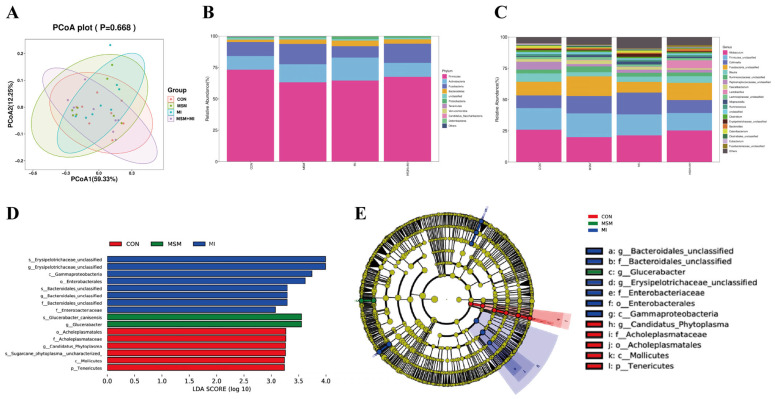
Fecal microbiota of poodles fed MSM- and MI-supplemented diets. (**A**) Principal coordinate analysis (PCoA) based on weighted UniFrac distances. (**B**) Fecal microbial communities predominant at the phylum level. (**C**) Fecal microbial communities predominant at the genus level. (**D**,**E**) LEfSe analysis. CON, basal diet; MSM, basal diet supplemented with 0.2% MSM; MI, basal diet supplemented with 0.2% MI; and MSM + MI, basal diet supplemented with 0.2% MSM and 0.2% MI.

Assessment of the fecal alpha diversity indices indicated that the number of observed species and the Chao1 indices were greater (*p* < 0.05) in the MI group than in the CON, MSM, and MSM + MI groups on Day 65 (Table 9). The PCoA plots revealed that similar microbial communities were present in the four groups (*p* > 0.05, Figure 5A).

The predominant fecal phyla in all poodles were Firmicutes, Actinobacteria, Fusobacteria, Bacteroidetes, Proteobacteria, Tenericutes, Verrucomicrobia, Candidatus Saccharibacteria, and Deferribacteres (Figure 5B). Poodles whose diets were supplemented with MSM and MI had significantly lower abundances of Tenericutes (Table 10). The most abundant groups were *Allobaculum*, *Firmicutes_unclassified*, *Collinsella*, *Fusobacteria_unclassified*, *Blautia*, *Peptostreptococcaceae_unclassified*, *Faecalibacterium*, *Lactobacillus*, and *Lachnospiraceae_unclassified* (Figure 5C). The poodles in the CON group presented significantly greater (*p* < 0.05) abundances of *Proteobacteria_unclassified* and *Candidatus Phytoplasma* than did those in the MSM, MI, and MSM + MI groups (Table 10), and the relative abundance of *Candidatus Phytoplasma* was greater in the MSM group than in the MI group (*p* < 0.05). The relative abundance of *Gammaproteobacteria_unclassified* was greater in the MSM and MI groups than in the CON group (*p* < 0.05). Compared with the CON group, the MSM group presented greater abundance of *Glucerabacter* (*p* < 0.05). Greater *Paramuribaculum* and *Hafnia* abundances were observed in the MSM group than in the CON and MSM + MI groups (*p* < 0.05). The abundance of *Enterobacter* and *Kineothrix* was greater (*p* < 0.05) in the MI group than in the CON and MSM + MI groups. Greater abundances (*p* < 0.05) of *Bacteroidales_unclassified*, *Halanaerobium*, *Mycobacterium*, and *Erysipelotrichaceae*_unclassified were observed in the MI group than in the CON, MSM, and MSM + MI groups.

LEfSe analysis was performed to identify microbial taxa that serve as biomarkers for the different groups. The results revealed that *Candidatus Phytoplasma* was strongly enriched in the CON group, *Glucerabacter* was enriched in the MSM group, and *Bacteroidales_unclassified* and *Erysipelotrichaceae_unclassified* were enriched in the MI group (Figure 5C,D).

### 3.5. Fecal Metabolomics

**Figure 6:**
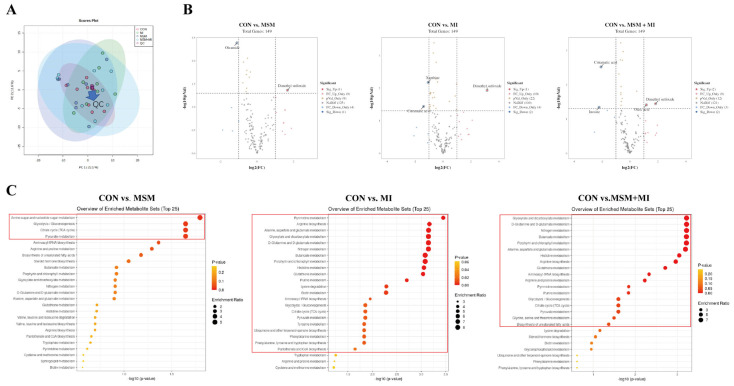
Fecal metabolomics of poodles fed MSM − and MI − supplemented diets. (**A**) Score plots from the PCA model among the three groups. (**B**) Volcano plot. (**C**) KEGG metabolic pathway enrichment analysis based on differential fecal metabolites. CON, basal diet; MSM, basal diet supplemented with 0.2% MSM; MI, basal diet supplemented with 0.2% MI; and MSM + MI, basal diet supplemented with 0.2% MSM and 0.2% MI.

The PCA score plots did not show clear separations among the groups (Figure 6A). A total of 149 metabolites were detected in feces. The changes in the metabolites are shown in Figure 6B. The differences in fecal metabolites among the four groups were screened using volcano plots, and fecal dimethyl sulfoxide was found to be upregulated in the MSM, MI, and MSM + MI groups (Figure 6B). In addition, fecal citramalic acid was downregulated in the MI and MSM + MI groups.

To gain further insight into the metabolic changes that occurred in the animals given dietary supplements, a KEGG pathway analysis of the differentially abundant metabolites was performed. As shown in Figure 6C and Table S1, carbohydrate metabolism (amino sugar and nucleotide sugar metabolism, glycolysis/gluconeogenesis, the citrate cycle (TCA cycle), and pyruvate metabolism) was the main pathway affected in the MSM group. MI supplementation significantly affected six metabolic pathways: carbohydrate metabolism (glyoxylate and dicarboxylate metabolism, butanoate metabolism, glycolysis/gluconeogenesis, the citrate cycle (TCA cycle), and pyruvate metabolism), nucleotide metabolism (pyrimidine metabolism, arginine biosynthesis, alanine, aspartate and glutamate metabolism, histidine metabolism, purine metabolism, lysine degradation, tyrosine metabolism, phenylalanine metabolism, phenylalanine, tyrosine and tryptophan biosynthesis), the metabolism of other amino acids (D-glutamine and D-glutamate metabolism and glutathione metabolism), energy metabolism (nitrogen metabolism), the metabolism of cofactors and vitamins (porphyrin and chlorophyll metabolism, biotin metabolism, ubiquinone and other terpenoid-quinone biosynthesis and pantothenate and CoA biosynthesis), and translation (aminoacyl-tRNA biosynthesis). The metabolic pathways most affected in the MSM + MI group were carbohydrate metabolism, nucleotide metabolism, metabolism of other amino acids, metabolism of cofactors and vitamins, translation, and lipid metabolism.

## 4. Discussion

MSM and MI might serve as functional ingredients in pet diets because of their beneficial effects on hair quality and their influence on the gut microbiota and the metabolome. In the present study, we used poodles as model animals to evaluate the effects of dietary supplementation with MSM and MI on physiological function, inflammatory cytokine levels, hair quality, gut microbiota, and metabolomics. All of the experimental diets had similar ingredient compositions and nutrient levels, with the only difference being the addition of MSM and MI. Supplementation with MSM and MI influenced hair quality and modulated the fecal microbiota and metabolic profiles of the poodles. Physiological function, inflammatory cytokine levels, and antioxidant capacity were not altered by MSM or MI supplementation, supporting the safety of including 0.2% MSM and/or MI in dog diets.

Poodles have an anagen-dominated coat type, similar to humans and sheep; approximately 98% of the follicles in poodles are in anagen [35]. However, in most dog breeds, the frequency of the telogen stage is significantly greater, and the percentage of telogen follicles can be as high as 34% [36]. Poodles were therefore a good choice for studying the effects of MSM and MI.

Supplementation of the diet with MSM or MI did not significantly affect hair length or hair score. A variety of factors, including nutritional imbalances, environmental factors and inflammatory diseases, can lead to excessive greasiness of the skin and hair [37]. To maintain desirable hair properties, understanding the physical and chemical structure of hair fibers is important. Hair consists of two separate structures: the hair shaft, which is visible on the body surface, and the follicle in the skin [38]. The hair shaft is composed of a cortex and cuticle cells [39]. The cuticle is most important in the study of hair quality because it comes into contact with combing devices, skin and other surfaces [40]. The cuticle plays a critical role in hair durability, felting, and shrinking [41]. In addition, the properties of the cuticle can maintain the hair in a clean and disentangled state, and they have a considerable impact on the hair’s appearance [42]. The thickness, height, and diameter of cuticle scales are correlated with the gloss, softness, and smoothness of hair [43]. A thicker scale corresponds to a higher friction coefficient and a sharper scale surface. A lower friction coefficient is related to softer and smoother hair [44]. In this study, the scale thickness on Day 65 was lower in the MI and MSM + MI groups than in the CON group, indicating that dietary supplementation with MI and MSM + MI decreased the friction coefficient and made the hair smoother. Compared with the scale thickness on Day 0, the scale thickness in the MSM + MI group on Day 65 decreased significantly, indicating that MSM + MI was more effective at making hair smoother than either MSM or MI alone. There were no significant differences among the four groups in the height or diameter of the hair.

Keratin is an important structural component of hair and is rich in sulfur-containing amino acids such as methionine and cysteine [45]. Owing to cysteine’s ability to form disulfide bonds, keratin has high strength and rigidity [46]. Research on wool has revealed that more lustrous wools contain more cysteine [47]. The synthesis of cysteine in the body requires a steady supply of methionine. A deficiency of methionine and cysteine can lead to hair loss, slow growth, and a brittle appearance of the hair [48]. In guinea pigs, the supplementation of a diet with radiolabeled MSM results in the incorporation of labeled sulfur into serum proteins containing methionine and cysteine, indicating that MSM may provide a source of sulfur [8]. Previous studies have shown that MSM may be metabolized to yield sulfur-containing proteins such as keratin, leading to increased sulfur content in the hair [49]. Recently, supersulfides and reactive sulfur were found to be present as intra- and extracellular components in mammals [50]. A study revealed that there was a high concentration of polysulfide, which imparts antioxidant activity and contributes to maintaining hair homeostasis, in the hair cuticle [51]. Our study revealed a significant increase in S levels in hair after the supplementation of the diet with MI or MSM + MI, indicating that the hair of the animals in these groups received more sulfur nourishment during the period from Day 0 to Day 65. Dietary supplements containing MI and MSM may provide a source of S for keratin or be metabolized to yield sulfur-containing compounds that act as hair supplements. In summary, dietary MI and MSM + MI, particularly MSM + MI, have beneficial effects on improving hair smoothness and increasing the sulfur content of hair.

Diet plays a crucial role in shaping the structure and function of the gut microbiome [52]. Gut microorganisms help maintain skin homeostasis by regulating systemic immunity and are key players in and regulators of the “gut–skin axis” [53]. The gut and the microbiota therein produce metabolites that can enter the circulation and modify the skin [54]. Unhealthy skin leads to compromised preemergent hair formation and poorly formed hair [55]. *Glucerabacter* efficiently hydrolyzes glucosylceramide and likely plays a major role in glucosylceramide hydrolysis in the poodle intestine [56]. Sphingoid bases metabolically derived from glucosylceramide are beneficial to hair because they enhance the formation and maintenance of an intact epidermal lipid barrier [57]. A relatively high relative abundance of *Glucerabacter* was detected in the fecal samples from poodles fed MI or MSM + MI, and this may have beneficial effects on the nourishment of hair.

In our study, changes in fecal metabolites were investigated via metabolomics based on the UPLC–Orbitrap–MS/MS analysis. The anti-inflammatory properties of dimethyl sulfoxide (DMSO) have great potential for supporting skin and hair health [58]. Dimethyl sulfoxide concentrations less than 1% significantly inhibited the growth of three strains of dermatophytes (*Trichophyton*, *Epidermophyton* and *Microsporum*), fungi that commonly cause skin, hair and nail infections [59]. Dimethyl sulfoxide appears to be safe for use in small doses [60]. At low concentrations (< 15 vol%), DMSO can enhance the permeability of lipid membranes and preserve cell integrity [61]. In the MSM, MI and MSM + MI groups, fecal dimethyl sulfoxide was significantly increased. Fecal dimethyl sulfoxide and its detrimental effects will require further exploration and verification. Carbohydrates are important sources of energy in the body and are linked to hair health. The carbohydrate pathway was affected in all three groups in which MI and MSM were added to the diets. Lipid metabolism is believed to play a crucial role in forming the lipid envelope of hair and skin [62]. Lipids are vital for improving hair glossiness, protecting against damage and maintaining healthy hair [63]. In the current study, supplementation of the diet with MSM + MI affected the lipid metabolism pathway. These results appear consistent with other studies suggesting that MI is involved in lipid signaling [64]. However, owing to the lack of studies on the metabolism of MSM and MI, further exploration and verification are needed to understand the associations between fecal metabolism and supplementation of the diet with MSM and MI.

However, there are some limitations in this study. We did not explore the effect of different doses in dogs. Our current date did not demonstrate the molecular mechanisms of the action of MSM and MI. Future clarification of these limitations will lead to the practical application of MSM and MI.

## 5. Conclusions

In conclusion, supplementation of the diet with MSM and MI enhanced hair smoothness in poodles, suggesting that such supplementation has a beneficial effect on hair quality. The microbiota and metabolomics analyses revealed that the addition of MSM (0.2%) and/or MI (0.2%) to the diet may have beneficial effects on the gastrointestinal health of poodles. All observed differences in serum biochemistry were within acceptable ranges, suggesting that poodles can maintain good health while being fed a diet containing MSM and MI. Further investigations that explore the underlying mechanism of the effects of MSM and MI on hair quality are needed.

## Figures and Tables

**Table 1:** Ingredients and nutrient levels of the experimental diet.

Items	Experimental Diet %
Ingredients (as-is basis, %)	
Chicken	18.00
Fish	15.00
Kelp powder	1.00
Chicken meat meal	12.00
Duck meat meal	10.00
Fish meat meal	6.00
Alfalfa granule	4.00
lactalbumin powder	3.50
Yucca	0.10
Rosemary	0.10
Plantago	0.10
Apple	0.20
Spinach	0.10
Carrot	0.10
Blueberry	0.10
Pumpkin seeds	0.20
Cranberry	0.10
Apple pear	0.20
Chicory root	0.20
Potato flour	6.00
Sweet potato flour	6.00
Beer yeast	2.00
Lysine	0.30
Vitamins and minerals premix ^1^	3.70
Solid flavoring agent	5.00
Fish oil	1.50
Chicken oil	4.50
Nutrient levels ^2^ (DM basis, %)	
DM	89.25
OM	88.50
CP	35.34
Fat	13.61
CF	9.31
GE, kcal/kg	4376.69
ME ^3^, kcal/kg	2663.29

^1^ The premix provided the following per kilogram of diet: vitamin A, 1,250,000 IU; vitamin D3, 120,000 IU; vitamin E, 12,000 mg; vitamin K3, 120 mg; vitamin B1 (thiamine), 500 mg; vitamin B2 (riboflavin), 400 mg; vitamin B6, 500 mg; vitamin B12, 8 mg; niacin, 2000 mg; calcium pantothenate, 1800 mg; biotin, 60 mg; folacin, 60 mg; choline, 4800 mg; Fe, 10,000 mg; Cu, 1200 mg; Mn, 4000 mg; Zn, 15,000 mg; I, 200 mg; Se, 20 mg. ^2^ Measured values. ^3^ The metabolizable energy was calculated via the following equation (Jewell and Jackson, 2023): Canine ME (Kcal/kg) = 48.7 + (0.934) × GE-(7.21) × Moisture − (50.19) × CP + (64.9) × Fat − (41.2) × CF − (13.4) × Ash + (0.00736) × GE × CP − (0.0126) × GE × Fat − (3.323) × CF × Ash [30]. DM, dry matter; OM, organic matter; CP, crude protein; Fat, acid-hydrolyzed fat; CF, crude fiber; GE, gross energy; and ME, metabolizable energy.

**Table 2:** Effects of MSM and MI on body weight, feed intake, and fecal output in poodles.

Item	Dietary Treatment ^1^				SEM	*p* Value
	CON	MSM	MI	MSM + MI		
Initial BW, kg	4.02	4.03	4.03	4.03	0.12	1.000
Final BW, kg	4.27	4.72	4.29	4.39	0.19	0.848
Average daily gain, g/d	3.85	10.58	4.14	5.18	1.78	0.530
Feed intake (as fed), g/d	98.20	115.79	100.23	94.63	6.15	0.651
Feed intake (DM), g/d	87.64	104.61	89.96	85.01	5.54	0.618
GE intake, kcal/d	383.58	469.24	400.31	380.84	26.78	0.574
Fecal output (as-is), g/d	55.50	59.83	50.83	53.38	4.29	0.908
Fecal output (DM), g/d	31.14	31.79	25.42	28.27	2.52	0.814

^1^ CON, basal diet; MSM, basal diet supplemented with 0.2% MSM; MI, basal diet supplemented with 0.2% MI; MSM + MI, basal diet supplemented with 0.2% MSM and 0.2% MI. BW, body weight; DM, dry matter.

**Table 3:** Effects of MSM and MI on nutrient digestibility in poodles (DM basis, %).

Item	Dietary Treatment ^1^				SEM	*p* Value
	CON	MSM	MI	MSM + MI		
DM	65.79	69.80	73.02	68.19	1.33	0.282
CP	67.25	74.16	76.57	71.73	1.46	0.129
Fat	89.01 ^b^	92.14 ^a^	91.98 ^a^	91.06 ^a,b^	0.44	0.035
CF	20.35	17.00	25.62	18.98	2.53	0.686
GE	76.86	79.98	77.85	81.64	1.01	0.346

^a,b^ Values in a row with no common superscripts differ significantly (*p* < 0.05). ^1^ CON, basal diet; MSM, basal diet supplemented with 0.2% MSM; MI, basal diet supplemented with 0.2% MI; MSM + MI, basal diet supplemented with 0.2% MSM and 0.2% MI. DM, dry matter; OM, organic matter; CP, crude protein; Fat, acid-hydrolyzed fat; CF, crude fiber; and GE, gross energy.

**Table 4:** Effects of MSM and MI on serum biochemical parameters in poodles.

Item	Range	Dietary Treatment ^1^				SEM	*p* Value
		CON	MSM	MI	MSM + MI		
D 0							
Albumin, g/L	23.00–40.00	33.10	33.79	33.68	33.50	0.55	0.976
TP, g/L	52.00–82.00	69.04	70.36	65.29	69.66	0.86	0.152
Globulin, g/L	23.00–45.00	35.95	36.56	31.63	36.15	0.94	0.215
A/G	0.80–2.00	0.94	0.95	1.09	0.96	0.04	0.444
AST, U/L	0.00–50.00	39.75	39.25	37.63	36.25	1.05	0.648
ALT, U/L	5.00–125.00	36.00	32.88	43.00	45.50	2.64	0.301
Amylase, U/L	400.00–1500.00	768.75	800.13	715.00	767.13	28.96	0.791
Creatinine, µmol/L	44.00–159.00	51.65	57.68	54.14	54.61	2.02	0.788
BUN, mmol/L	2.50–9.60	5.76	6.18	5.70	6.48	0.33	0.834
BUN/CREA	16.00–218.00	119.77	107.77	110.31	115.81	5.51	0.880
Glucose, mmol/L	4.11–7.94	4.19 ^b^	3.91 ^b^	4.53 ^a,b^	5.01 ^a^	0.14	0.028
D 65							
Albumin, g/L	23.00–40.00	34.66 ^b^	40.60 ^a^	35.13 ^b^	33.95 ^b^	0.80	0.006
TP, g/L	52.00–82.00	72.30 ^b^	83.38 ^a^	71.36 ^b^	71.55 ^b^	1.70	0.023
Globulin, g/L	23.00–45.00	37.63	42.79	36.24	37.61	1.36	0.348
A/G	0.80–2.00	0.94	0.99	1.01	0.94	0.04	0.885
AST, U/L	0.00–50.00	55.13	56.38	50.75	50.50	1.77	0.562
ALT, U/L	5.00–125.00	42.63	39.63	45.50	37.75	2.32	0.677
Amylase, U/L	400.00–1500.00	672.75	820.13	723.00	660.38	39.59	0.490
Creatinine, µmol/L	44.00–159.00	57.75	68.33	62.76	65.74	3.05	0.667
BUN, mmol/L	2.50–9.60	6.95	7.33	7.17	6.67	0.33	0.916
BUN/CREA	16.00–218.00	127.66	108.07	120.06	107.19	5.86	0.566
Glucose, mmol/L	4.11–7.94	3.39	4.12	4.16	3.97	0.21	0.570

^a,b^ Values in a row with no common superscripts differ significantly (*p* < 0.05). ^1^ CON, basal diet; MSM, basal diet supplemented with 0.2% MSM; MI, basal diet supplemented with 0.2% MI; MSM + MI, basal diet supplemented with 0.2% MSM and 0.2% MI. TP, total protein; A/G, albumin/globulin; AST, aspartate aminotransferase; ALT, alanine aminotransferase; CK, creatine kinase; CREA, creatinine; BUN, blood urea nitrogen; and BUN/CREA, blood urea nitrogen/creatinine.

**Table 5:** Effects of MSM and MI on inflammatory cytokine levels in poodles.

Item	Dietary Treatment ^1^				SEM	*p* Value
	CON	MSM	MI	MSM + MI		
Interferon-γ (ng/L)	26.53	26.79	27.91	27.33	0.43	0.682
Interleukin-1β (ng/L)	83.72	89.52	77.59	97.76	5.30	0.633
Immunoglobin G (μg/mL)	131.43	169.02	175.80	124.29	13.06	0.425
Interleukin-8 (pg/mL)	240.33	241.85	247.32	254.73	4.67	0.747
Tumor necrosis factor (pg/mL)	63.12	61.79	63.16	68.39	1.80	0.658

^1^ CON, basal diet; MSM, basal diet supplemented with 0.2% MSM; MI, basal diet supplemented with 0.2% MI; and MSM + MI, basal diet supplemented with 0.2% MSM and 0.2% MI.

**Table 6:** Effects of MSM and MI on hair length (mm) in poodles.

Item	Dietary Treatment ^1^				SEM	*p* Value
	CON	MSM	MI	MSM + MI		
D 65	3.43	2.98	3.02	2.96	0.16	0.722

^1^ CON, basal diet; MSM, basal diet supplemented with 0.2% MSM; MI, basal diet supplemented with 0.2% MI; and MSM + MI, basal diet supplemented with 0.2% MSM and 0.2% MI.

**Table 7:** Effects of MSM and MI on the hair score of poodles on Day 65.

Item	Dietary Treatment ^1^				SEM	*p* Value
	CON	MSM	MI	MSM + MI		
Hair score	3.02	2.89	2.98	3.11	0.07	0.733
Ideal percentage (HS = 3), %	25.00%	26.79%	33.93%	48.21%	4.77	0.321
Greasy percentage (HS = 4 or 5), %	35.71%	33.93%	30.36%	30.36%	3.87	0.948
Dry percentage (HS = 1 or 2), %	39.29%	39.29%	35.71%	21.43%	3.66	0.280

^1^ CON, basal diet; MSM, basal diet supplemented with 0.2% MSM; MI, basal diet supplemented with 0.2% MI; and MSM + MI, basal diet supplemented with 0.2% MSM and 0.2% MI. The samples were scored according to the following scale: 1 = dull, coarse, dry; 2 = medium soft, medium dry; 3 = very soft, normal dry; 4 = medium knots, medium greasy; and 5 = severe knots, very greasy. A score of 1 or 2 was considered to indicate “dry”; a score of 3 was considered “ideal”; and a score of 4 or 5 was considered to indicate “greasy”.

**Table 8:** Effects of MSM and MI on the S content (%) of the hair of poodles.

Dietary Treatment ^1^	D 0	D 65	SEM	*p* Value
CON	9.06	7.97	0.67	0.124
MSM	9.52	10.26	1.36	0.601
MI	8.29 ^b^	10.31 ^a^	0.64	0.007
MSM + MI	7.50 ^b^	10.70 ^a^	1.08	0.018

^a,b^ Values in a row with no common superscripts differ significantly (*p* < 0.05). ^1^ CON, basal diet; MSM, basal diet supplemented with 0.2% MSM; MI, basal diet supplemented with 0.2% MI; and MSM + MI, basal diet supplemented with 0.2% MSM and 0.2% MI.

**Table 9:** Effects of MSM and MI on the alpha diversity of the fecal microbiota in poodles.

Item	Dietary Treatment ^1^				SEM	*p* Value
	CON	MSM	MI	MSM + MI		
Observed_species	197.38 ^b^	209.25 ^b^	333.88 ^a^	232.75 ^b^	15.77	0.009
Shannon	4.21	4.26	4.80	4.33	0.11	0.220
Simpson	0.87	0.87	0.89	0.86	0.01	0.510
Chao1	198.68 ^b^	210.83 ^b^	334.47 ^a^	233.61 ^b^	15.76	0.009

^a,b^ Values in a row with no common superscripts differ significantly (*p* < 0.05). ^1^ CON, basal diet; MSM, basal diet supplemented with 0.2% MSM; MI, basal diet supplemented with 0.2% MI; and MSM + MI, basal diet supplemented with 0.2% MSM and 0.2% MI.

**Table 10:** Effects of MSM and MI on the species present in fecal microbial communities (basis on relative abundance (% of sequences)) in poodles.

Item	Dietary Treatment ^1^				SEM	*p* Value
	CON	MSM	MI	MSM + MI		
Phylum						
Tenericutes	0.4075 ^a^	0.2113 ^b^	0.0638 ^b^	0.0613 ^b^	0.0339	0.005
Genus						
*Proteobacteria (unclassified)*	0.1600 ^a^	0.0563 ^b^	0.0025 ^b^	0.0188 ^b^	0.0142	0.008
*Candidatus_Phytoplasma*	0.3725 ^a^	0.1813 ^b^	0.0050 ^c^	0.0225 ^b.c^	0.0341	0.005
*Gammaproteobacteria (unclassified)*	0.0000 ^b^	0.0525 ^a^	0.0600 ^a^	0.0375 ^a,b^	0.0069	0.002
*Glucerabacter*	0.2300 ^b^	0.9188 ^a^	0.2450 ^a,b^	0.9013 ^a,b^	0.1107	0.003
*Paramuribaculum*	0.0013 ^b^	0.0213 ^a^	0.0113 ^a,b^	0.0063 ^b^	0.0022	0.013
*Hafnia*	0.0000 ^b^	0.0225 ^a^	0.0088 ^a,b^	0.0000 ^b^	0.0028	0.006
*Enterobacter*	0.0013 ^b^	0.0250 ^a,b^	0.0550 ^a^	0.0138 ^b^	0.0060	0.013
*Kineothrix*	0.0000 ^b^	0.0100 ^a,b^	0.1438 ^a^	0.0000 ^b^	0.0217	0.000
*Bacteroidales (unclassified)*	0.0238 ^b^	0.1075 ^b^	0.3863 ^a^	0.1113 ^b^	0.0444	0.005
*Halanaerobium*	0.0000 ^b^	0.0000 ^b^	0.0700 ^a^	0.0038 ^b^	0.0094	0.012
*Mycobacterium*	0.0000 ^b^	0.0000 ^b^	0.0088 ^a^	0.0000 ^b^	0.0013	0.022
*Erysipelotrichaceae (unclassified)*	0.8925 ^b^	0.6838 ^b^	2.4475 ^a^	0.7163 ^b^	0.2328	0.036

^a,b^ Values in a row with no common superscripts differ significantly (*p* < 0.05). ^1^ CON, basal diet; MSM, basal diet supplemented with 0.2% MSM; MI, basal diet supplemented with 0.2% MI; and MSM + MI, basal diet supplemented with 0.2% MSM and 0.2% MI.

## Data Availability

Data are contained within the article and Supplementary Materials.

## Supplementary Materials

The following supporting information can be downloaded at: https://www.mdpi.com/article/10.3390/ani14243643/s1, Table S1: KEGG enrichment analysis of differential serum metabolites among the groups.
