# Supplementary material for: Dietary Supplementation with Methylsulfonylmethane and Myo-Inosito Supports Hair Quality and Fecal Microbiome in Poodles
Source: Animals (Basel). 2024 Dec 17;14(24):3643. doi: 10.3390/ani14243643 (PMC11672428; doi:10.3390/ani14243643)
Supplement: Supplementary file 1 [file animals-14-03643-s001.zip › animals-3341245-supplementary.pdf]

Table S1. KEGG enrichment analysis of differential serum metabolites among the groups[a]

	Super pathway	Sub pathway	Differential metabolites	*P*-value
CON vs. MSM	Carbohydrate metabolism	Amino sugar and nucleotide sugar metabolism	N-Acetylneuraminic acid (↑)	0.016
		Glycolysis / Gluconeogenesis	Pyruvic acid (↑)	0.023
		Citrate cycle (TCA cycle)		0.023
		Pyruvate metabolism		0.023
CON vs. MI	Carbohydrate metabolism	Glyoxylate and dicarboxylate metabolism	L-Glutamic acid (↑)<br>Pyruvic acid (↑)<br>L-Glutamine (↑)	0.00071
		Butanoate metabolism	L-Glutamic acid (↑)	0.00083
		Glycolysis / Gluconeogenesis	Pyruvic acid (↑)	0.014
		Citrate cycle (TCA cycle)		0.014
		Pyruvate metabolism		0.014
	Nucleotide metabolism	Pyrimidine metabolism	L-Glutamine (↑)<br>Uridine (↑)<br>Cytidine (↑)<br>Thymine (↑)<br>Uracil (↑)	0.00035
		Arginine biosynthesis	L-Glutamic acid (↑)<br>L-Arginine (↑)<br>N-Acetylornithine (↑)<br>Citrulline (↑)<br>Ornithine (↑)<br>L-Glutamine (↑)	0.00068
		Alanine, aspartate and glutamate metabolism	D-Aspartic acid (↑)<br>L-Glutamic acid (↑)<br>L-Glutamine (↑)<br>Pyruvic acid (↑)	0.00071
		Histidine metabolism	L-Glutamic acid (↑) Carnosine (↑)<br>Methylimidazoleacetic acid (↑)	0.00088
		Purine metabolism	Xanthine (↑)<br>L-Glutamine (↑) Adenosine (↑) Deoxyinosine (↑) Hypoxanthine (↑)<br>Inosine (↑)<br>Guanine (↑)<br>Adenine (↑) Deoxyadenosin (↑)	0.002
		Lysine degradation	L-Lysine (↑)<br>N6, N6, N6-Trimethyl-L-lysine (↑)<br>Saccharopine (↑)<br>L-Pipecolic acid (↑)<br>L-Carnitine (↑)	0.005
		Tyrosine metabolism	L-Tyrosine (↑)<br>Pyruvic acid (↑)<br>Gentisic acid (↑)	0.015
		Phenylalanine metabolism	L-Phenylalanine (↑)	0.015
		Phenylalanine, tyrosine and tryptophan biosynthesis	L-Tyrosine (↑)	0.015
	Metabolism of other amino acids	D-Glutamine and D-glutamate metabolism	L-Glutamic acid (↑)<br>L-Glutamine (↑)	0.00071
		Glutathione metabolism	L-Glutamic acid (↑) Pyroglutamic acid (↑)	0.00091

			Ornithine (↑)<br>Spermidine (↑)	
	Energy metabolism	Nitrogen metabolism	L-Glutamic acid (↑)<br>L-Glutamine (↑)	0.00071
	Metabolism of co-factors and vitamins	Porphyrin and chlorophyll metabolism	L-Glutamic acid (↑)	0.00083
		Biotin metabolism	L-Lysine (↑)	0.005
		Ubiquinone and other terpe-noid-quinone biosynthesis	L-Tyrosine (↑)	0.015
		Pantothenate and CoA bio-synthesis	Pantothenic acid (↑)<br>L-Valine (↑)<br>Uracil (↑)	0.023
	Translation	Aminoacyl-tRNA biosynthe-sis	L-Phenylalanine (↑)<br>L-Arginine (↑)<br>L-Glutamine (↑)<br>L-Methionine (↑)<br>L-Valine (↑)<br>L-Lysine (↑)<br>L-Isoleucine (↑)<br>L-Leucine (↑)<br>L-Tryptophan (↑)<br>L-Tyrosine (↑)<br>L-Proline (↑)<br>L-Glutamic acid (↑)	0.011
CON vs. MSM+MI	Carbohydrate me-tabolism	Glyoxylate and dicarboxylate metabolism	L-Glutamic acid (↑)<br>Pyruvic acid (↑)<br>L-Glutamine (↑)	0.00060
		Butanoate metabolism	L-Glutamic acid (↑)	0.00061
		Glycolysis / Gluconeogenesis	Pyruvic acid (↑)	0.025
		Citrate cycle (TCA cycle)		0.025
		Pyruvate metabolism		0.025
	Nucleotide metabo-lism	Alanine, aspartate and glu-tamate metabolism	D-Aspartic acid (↑)<br>L-Glutamic acid (↑)<br>L-Glutamine (↑)<br>Pyruvic acid (↑)	0.00064
		Histidine metabolism	L-Glutamic acid (↑)<br>Carnosine (↑)<br>Methylimidazoleacetic acid (↑)	0.00090
		Arginine biosynthesis	L-Glutamic acid (↑)<br>L-Arginine (↑)<br>N-Acetylornithine (↑)<br>Citrulline (↑)<br>Ornithine (↑)<br>L-Glutamine (↑)	0.001
		Arginine and proline metabo-lism	L-Arginine (↑)<br>Creatine (↑)<br>Spermidine (↑)<br>N-Acetylputrescine (↑)<br>L-Proline (↑)<br>cis-4-Hydroxy-D-proline (↑)<br>L-Glutamic acid (↑)<br>Ornithine (↑)	0.006

	Purine metabolism	4-Guanidinobutanoic acid (↑)<br>Pyruvic acid (↑)<br>Xanthine (↑)<br>L-Glutamine (↑)<br>Adenosine (↑)<br>Deoxyinosine (↑)<br>Hypoxanthine (↑)<br>Inosine (↑)<br>Guanine (↑)<br>Adenine (↑)<br>Deoxyadenosin (↑)	0.014
	Pyrimidine metabolism	L-Glutamine (↑)<br>Uridine (↑)<br>Cytidine (↑)<br>Thymine (↑)<br>Uracil (↑)	0.014
	Glycine, serine and threonine metabolism	Choline (↑)<br>Creatine (↑)<br>Pyruvic acid (↑)	0.032
Metabolism of other amino acids	D-Glutamine and D-glutamate metabolism	L-Glutamic acid (↑)<br>L-Glutamine (↑)	0.00060
	Glutathione metabolism	L-Glutamic acid (↑)<br>Pyroglutamic acid (↑)<br>Ornithine (↑)<br>Spermidine (↑)	0.002
Energy metabolism	Nitrogen metabolism	L-Glutamic acid (↑)<br>L-Glutamine (↑)	0.00060
Metabolism of co-factors and vitamins	Porphyrin and chlorophyll metabolism	L-Glutamic acid (↑)	0.00061
Translation	Aminoacyl-tRNA biosynthesis	L-Phenylalanine (↑)<br>L-Arginine (↑)<br>L-Glutamine (↑)<br>L-Methionine (↑)<br>L-Valine (↑)<br>L-Lysine (↑)<br>L-Isoleucine (↑)<br>L-Leucine (↑)<br>L-Tryptophan (↑)<br>L-Tyrosine (↑)<br>L-Proline (↑)<br>L-Glutamic acid (↑)	0.005
Lipid metabolism	Biosynthesis of unsaturated fatty acids	Oleic acid (↑)<br>Linoleic acid (↑)<br>Docosahexaenoic acid (↑)	0.043

[a] KEGG, Kyoto Encyclopedia of Genes and Genomes.
CON, basal diet group; MSM, basal diet supplemented with 0.2% MSM group; MI, basal diet supplemented with 0.2% MI group; MSM + MI, basal diet supplemented with 0.2% MSM and 0.2% MI group.
